# Cardiac Pre-tamponade Secondary to Generalized Myxedema Due to Neglected Hashimoto’s Thyroiditis in a Child With Down Syndrome

**DOI:** 10.7759/cureus.60367

**Published:** 2024-05-15

**Authors:** Nadia Echcharii, Nabila Chekhlabi, Nezha Dini

**Affiliations:** 1 Pediatric Department, Cheikh Khalifa International University Hospital, Mohammed VI University of Health Science, Casablanca, MAR

**Keywords:** down syndrome, pre-tamponade, hashimoto, myxedema, child

## Abstract

Down syndrome (DS) is the most common chromosomal disorder in live-born infants, often associated with intellectual disability and various medical conditions, including thyroid dysfunction. Hashimoto's thyroiditis (HT), an autoimmune subtype, is a leading cause of acquired hypothyroidism in DS children. Severe hypothyroidism can precipitate myxedema, a critical condition linked to complications like pericardial effusion and cardiac tamponade. This case study presents a nine-year-old male with DS who was admitted for acute respiratory distress exhibiting classic signs of myxedema. Initial investigations revealed severe hypothyroidism and significant pericardial effusion. Surgical pericardiotomy drained 800 mL of fluid, confirming myxedema secondary to HT. Levothyroxine therapy led to progressive improvement, resolving myxedematous infiltrate and associated symptoms within a month. Follow-up at 12 months demonstrated sustained improvement with normalized thyroid function and no clinical disease activity. This case highlights an atypical presentation of HT in a DS child with cardiac pre-tamponade.

## Introduction

Down syndrome (DS) stands as the most prevalent chromosomal disorder among live-born infants and remains the leading cause of severe learning disabilities. A characteristic feature observed in children with DS is their increased susceptibility to various autoimmune diseases [1]. Among these, thyroid dysfunction is the most common, estimated to affect 4%-18% of children with DS [1]. The spectrum of thyroid dysfunction in DS patients encompasses congenital hypothyroidism, subclinical hypothyroidism, acquired hypothyroidism (both autoimmune and non-autoimmune), and hyperthyroidism [2].

Acquired hypothyroidism, often triggered by autoimmune thyroiditis, tends to manifest in older children. Hashimoto’s thyroiditis (HT), a subtype of autoimmune thyroiditis, typically presents with gradual, nonspecific symptoms of hypothyroidism that may go unnoticed for an extended period. The progression to myxedema is a rare occurrence, constituting a medical emergency with high mortality rates [3]. Consequently, pericardial effusion, a condition frequently associated with myxedema, is more prevalent in children with DS, particularly in cases of generalized myxedema that represents a severe stage of hypothyroidism. Notably, cardiac tamponade as the initial presentation of hypothyroidism is exceedingly rare [4]. In this context, we report a case involving a nine-year-old male, who experienced neglected HT, demonstrating an unusual presentation with cardiac pre-tamponade in the context of DS.

## Case presentation

A nine-year-old male with DS presented to the emergency department with acute respiratory distress. He is a second live birth from a non-consanguineous marriage and had a history of hypospadias surgery. He had been experiencing progressive dyspnea, asthenia, sluggishness, constipation, facial swelling, and muscle aches over several weeks.

**Figure 1:**
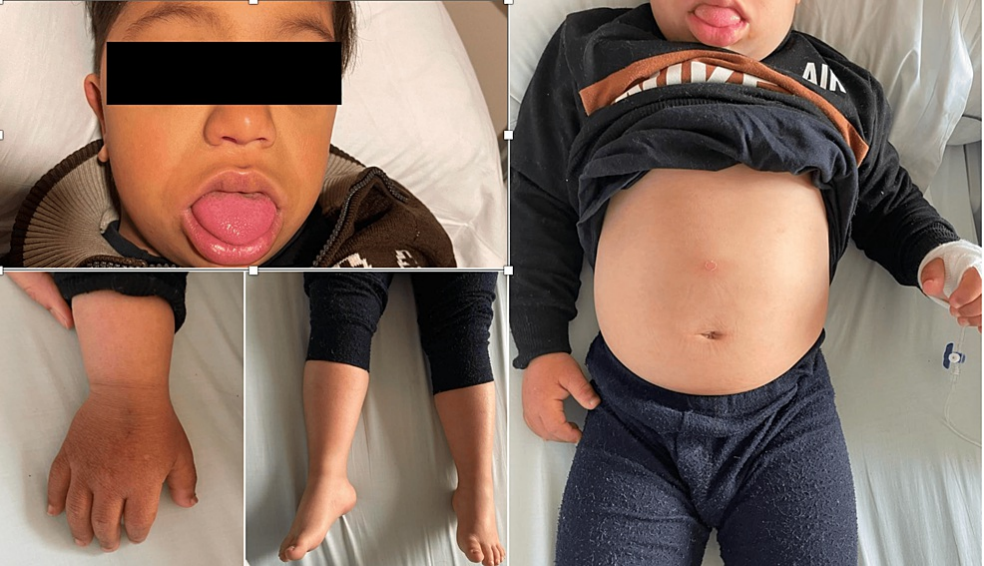
Generalized puffiness associated with myxedema.

Upon admission, the patient exhibited respiratory distress (oxygen saturation <85%, respiratory rate 45 breaths/min, heart rate 60 beats/min, and blood pressure 75/40 mmHg), along with signs of myxedematous infiltration, puffy face, decreased muscle tone, macroglossia, abdominal swelling, and edema of the lower limbs. The patient's body temperature was 36.8°C. Notable physical features included dry skin, diffuse myxedematous infiltration (Figure 1), and a thyroid gland difficult to palpate due to neck edema. Heart sounds were muffled, and peripheral pulses were normal.

Initial investigations revealed increased serum creatinine levels (0.87 mg/dL), creatinine clearance of 51 mL/min, urea concentration of 0.3 g/L, and albumin rate of 43 g/L (normal range 9.8-16.3). Complete blood count showed hemoglobin of 10.8 g/dL, white blood cell count of 5.9 × 10^3/μL, and platelet count of 277 × 10^3/μL. Thyroid function tests indicated severe hypothyroidism with an elevated thyroid-stimulating hormone (TSH) level of 845.6 μIU/mL (normal range 0.4-4.4), low levels of FT3 (0.3 pmol/L; normal range 9.8-16.3), and FT4 (0.388 pmol/L; normal range 9.8-16.3), along with highly positive anti-thyroid peroxidase antibodies (1,796.5 U/mL; normal value < 34) and an anti-thyroglobulin level of 359 µg/L (normal value < 25 µg/L).

**Figure 2:**
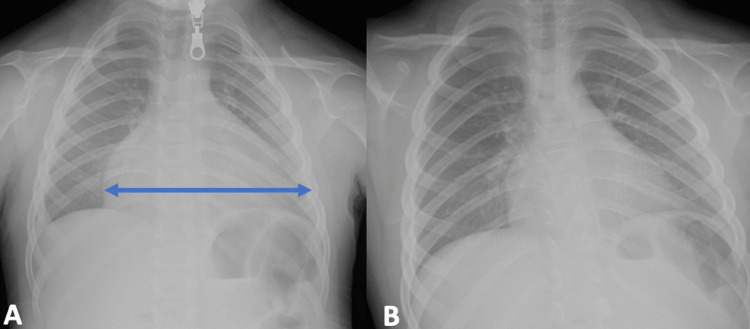
(A) Chest radiograph showing cardiomegaly with globular enlargement of the cardiac silhouette. (B) Chest radiograph, taken after six months, revealing no evidence of cardiomegaly.

**Figure 3:**
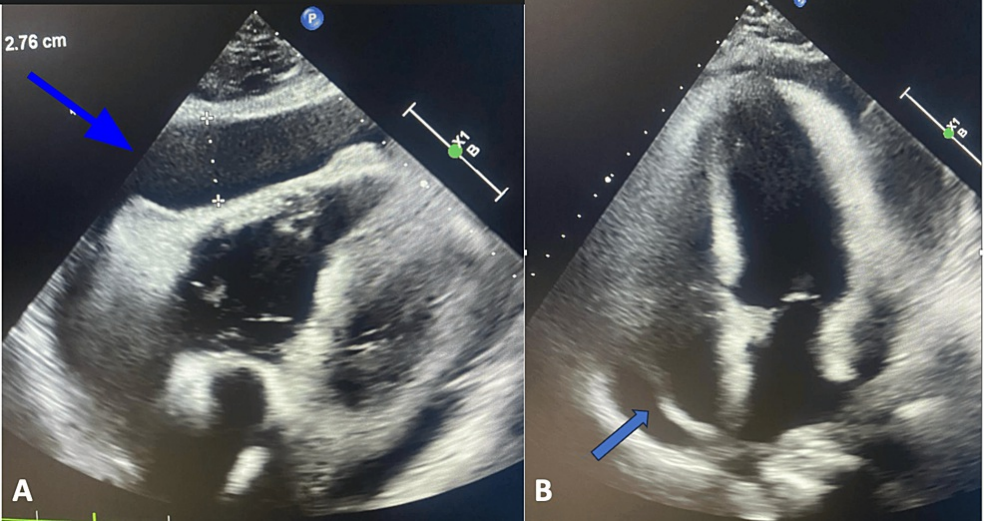
(A) Pericardial effusion adjacent to the right heart chambers measuring 27 mm. (B) Four-chamber slice showing a pericardial effusion adjacent to the right chambers.

Chest radiography revealed an enlarged cardiac silhouette with a cardiothoracic ratio of 0.7 (Figures 2A, 2B). Transthoracic echocardiography identified significant pericardial effusion compressing the right cardiac chambers, while the left ventricular ejection fraction remained within the normal range (Figures 3A, 3B). An electrocardiogram initially showed sinus bradycardia with a prolonged QT segment. Abdominal ultrasound showed a small amount of peritoneal effusion. Ultrasonography of the thyroid revealed diffuse hypoechogenicity of the thyroid gland. The surgical pericardiotomy yielded 800 mL of serous, yellowish-clear pericardial fluid.

The clinical and laboratory findings suggested a diagnosis of myxedema secondary to severe hypothyroidism (Hashimoto's disease). Replacement therapy with levothyroxine was initiated at an initial dosage of 50 μg/day for four days, followed by upward titration of 25 μg every four days, reaching a final dose of 150 μg. The patient is currently showing improvement with a progressive normalization of thyroid function.

After one month of treatment, there was a notable disappearance of the myxedematous infiltrate and drowsiness. The patient showed no cardiac or respiratory signs, and there was a resumption of normal activities. A follow-up biochemical analysis at three months revealed a decrease in TSH levels to 4.54 μUI/mL, along with an increase in FT3 (7.46 pmol/L) and FT4 (2.43 pmol/L). Additionally, the creatinine level decreased to 0.5 mg/dL.

During the latest follow-up conducted 12 months after the diagnosis of this disease, the patient exhibited no clinical signs of the disease, and thyroid function remained normal. Written consent was obtained from the parents.

## Discussion

In the case of DS, our patient gradually experienced constipation, drowsiness, and chronic asthenia; however, these symptoms went unnoticed. It can be assumed that a very gradual loss of thyroid function allowed better tolerance to hypothyroidism. Subsequently, the child decompensated with myxedema accompanied by severe pericardial effusion, complicated by pre-tamponade and a transient elevation of serum creatinine. Investigations revealed hypothyroidism, with a T4 level of almost 0.388 pmol/L and a TSH value above 845.6 μIU/mL.

DS is the most common chromosomal condition, occurring in approximately one of every 600-800 live births. DS patients typically exhibit facial dysmorphisms, intellectual disability, and multiple medical conditions, including cardiac, pulmonary, and gastrointestinal anomalies, as well as an increased risk of hematologic malignancies and immunological disorders. Endocrine disorders, such as thyroid dysfunction, diabetes, and short stature, are much more prevalent than in the general population [5-7]. Thyroid dysfunction, ranging from hypothyroidism to hyperthyroidism, either overt or subclinical, of autoimmune or non-autoimmune etiology, is particularly common in DS [8].

Children with DS are at greater risk of autoimmune thyroid diseases, with HT being the most common autoimmune disease. Its prevalence is believed to be much higher in DS patients than in the general population of the same age [9,10]. Hashimoto’s disease, the most common cause of hypothyroidism, is diagnosed by the presence of anti-thyroid peroxidase and anti-thyroglobulin antibodies [7].

Untreated hypothyroidism can affect many organs and may be asymptomatic, but its complications can be fatal. Common symptoms in children include constipation, lethargy, stunted growth, fatigue, swelling of the face, dry skin with non-pitting edema, and delayed relaxation of deep tendon reflexes [9]. Complications may include myxedema, characterized by non-focal thickening and induration of the skin due to increased deposition of connective tissue constituents leading to fluid retention and swelling of subcutaneous tissues. Profoundly decompensated hypothyroidism can lead to myxedema coma, characterized by altered mental status and changes in vital signs such as hypothermia, bradycardia, and hypotension, representing a medical emergency [11]. Additionally, increased serum creatinine is frequently reported in hypothyroid patients and may be due to transient functional renal failure [12]. Pericardial effusion, a rare complication, is caused by increased capillary permeability and disruption of lymphatic drainage. The volume of the pericardial effusion is related to the duration and severity of hypothyroidism and may present as an inaugural tamponade or a pre-tamponade [13].

Myxedematous tamponade is a major medical emergency. The initial treatment involves drainage of pericardial fluid through pericardiocentesis and/or pericardiostomy, followed by hormone replacement therapy with levothyroxine [14]. During levothyroxine treatment, it is noteworthy that the resolution of cardiovascular pericardial effusions may commence even prior to achieving euthyroidism, highlighting the early benefits of therapy on cardiovascular health. Complete resolution of the effusion typically occurs between eight and 26 weeks [14,15]. Our patient benefited from pericardiostomy followed by oral replacement treatment with levothyroxine immediately after the diagnosis of hypothyroidism, showing good progress.

## Conclusions

This case highlights the significance of recognizing and managing thyroid dysfunction in individuals with DS, particularly HT. Early detection and treatment are crucial to prevent severe complications such as pericardial effusion. Levothyroxine replacement therapy and a multidisciplinary approach play pivotal roles in improving outcomes for these patients. Continued research and clinical vigilance are essential for optimizing care and enhancing the quality of life for individuals with DS.
